# Georgia Medical Association

**Published:** 1872-03

**Authors:** 


### EDITORIAL AND MISCELLANEOUS.

### GEORGIA MEDICAL ASSOCIATION.

As announced by the Permanent Secretary, the Medical Association of the State will assemble in the city of Columbus on the second Wednesday of April (10th proximo.) We would earnestly urge a general attendance upon the part of the profession throughout the State. We know that some of our best men have ceased to take any interest in this annual meeting, on account of the controversies which have interfered with its proceedings to such an extent as to pervert it entirely from its original and proper design as a scientific body, resulting in great discredit not only to the organization, but to the entire profession of the State. Without reference to the right or the wrong of the contending parties, and without assuming any partisanship whatever, the last meeting of the body, by an overwhelming majority, decided that this discreditable strife should never have been introduced into it—having assumed essentially from the first (whatever admixture of honest contention for truth or right may have characterized some individuals) a bitter personal character, entirely foreign to the prosecution of science, and antagonistic to the honor and dignity of such an organization.

So far as we know or believe, this action of the last meeting will be sustained by the meeting at Columbus; and we do not think there is presumption in giving the assurance that no further discussion in reference to the exciting questions referred to will be permitted within the Association; otherwise, we could with no propriety ask for a general attendance, and indeed should not regard the continued existence of the body as in any way advantageous to the interests of medical science.

Under its present management,* this journal has studiously avoided any reference to the *merits* of the controversies alluded to, and does not now propose to indulge in any partial or partisan censure of any individual who may have been a party to the contest, but has no hesitation in asserting that the Georgia Medical Association has no interest in any conflict which its individual members may choose to perpetuate, except in protecting its own dignity and character by refusing it an entrance into the deliberations of the body.

However dissatisfied any individual may have been heretofore with the action of the last Association, which refused to act in the interest of partisanship, it is certainly to be hoped that a sense of what is due to the voice of that body, and to the profession of the city of Columbus, who have taken no part in these controversies, as well as what is due to science, harmony and common decency, will prevent the attempt to make the meeting there the occasion for a renewal of the *strife* which has traveled, year after year, the rounds of the Association—making it a "stench in the nostrils" of the profession and the people wherever it appeared—and which, to its honor was so decidedly ejected at the last meeting in Americus.

We hear of papers in course of preparation, to be presented at the Columbus meeting, and we sincerely hope that the prediction of a distinguished member of the body will be realized—namely, that there will be larger contributions to science upon that, than upon any similar occasion for the last ten years.

We cannot close this hasty article without earnestly urging those who have been antagonists (in the unfortunate difficul-

---

* Since the connection of the author of this article and the present Senior Editor of the JOURNAL, who has refused to discuss the *merits* of what he has honestly regarded as a *personal* controversy, originating in and essentially belonging to Atlanta—to which he was no party, and in which he had no interest, save in excluding it from the Association—and this, too, in the face of the violent and unprovoked assault made upon him at the last meeting of the Association, and the persistent attempts to engage him in the controversy since, by publications which have only served to discredit their authors, to demonstrate the true nature of the contest, and to establish beyond cavil the correctness of the action at Americus.—J. P. L.

ties which all must admit have been suicidal in their character to the best interests of individuals and the whole profession of the State) to meet at Columbus—not to attempt to revive the old controversies, but with the determined purpose to forgive and forget, and to repair, as rapidly as possible, the damaged reputation of the medical profession of Georgia. With such a spirit of conciliation and concession as should, and we hope will prevail, not only all differences (if such still exist) between gentlemen, may be satisfactorily arranged *outside* of the Association, but, as we honestly believe, a spirit of harmony may be inaugurated, which will introduce an era of good feeling into the Association which has been unknown for nearly twenty years.

